# The Synthesis of B-Doped Porous Carbons via a Sodium Metaborate Tetrahydrate Activating Agent: A Novel Approach for CO_2_ Adsorption

**DOI:** 10.3390/molecules30122564

**Published:** 2025-06-12

**Authors:** Junting Wang, Yingyi Wang, Xiaohan Liu, Qiang Xiao, Muslum Demir, Mohammed K. Almesfer, Suleyman Gokhan Colak, Linlin Wang, Xin Hu, Ya Liu

**Affiliations:** 1 Key Laboratory of the Ministry of Education for Advanced Catalysis Materials, Zhejiang Normal University, Jinhua 321004, China; wwwjting@163.com (J.W.); wangyingyi0@126.com (Y.W.); 19548990836@163.com (X.L.); 2 Institute of Plant Nutrition, Resources and Environment, Beijing Academy of Agriculture and Forestry Science, Beijing 100097, China; xqiang1978@163.com; 3 Department of Chemical Engineering, Bogazici University, Istanbul 34342, Türkiye; demirm@alumni.vcu.edu; 4 TUBITAK Marmara Research Center, Material Institute, Gebze 41470, Türkiye; 5 Chemical Engineering Department, College of Engineering, K. S. A & Central Labs, King Khalid University, Abha 61413, Saudi Arabia; almesfer@kku.edu.sa; 6 Department of Biomedical Engineering, Faculty of Engineering and Natural Sciences, Iskenderun Technical University, Hatay 31200, Türkiye; sgokhan.colak@iste.edu.tr; 7 Key Laboratory of Urban Rail Transit Intelligent Operation and Maintenance Technology and Equipment of Zhejiang Province, College of Engineering, Zhejiang Normal University, Jinhua 321004, China; wanglinlin@zjnu.cn

**Keywords:** B-doped porous carbons, CO_2_ adsorption, biomass, sodium metaborate tetrahydrate

## Abstract

The CO_2_ capture from flue gas using biomass-derived porous carbons presents an environmentally friendly and sustainable strategy for mitigating carbon emissions. However, the conventional fabrication of porous carbons often relies on highly corrosive activating agents like KOH and ZnCl_2_, posing environmental and safety concerns. To address this challenge, in the present work sodium metaborate tetrahydrate (NaBO_2_·4H_2_O) has been utilized as an alternative, eco-friendly activating agent for the first time. Moreover, a water chestnut shell (WCS) is used as a sustainable precursor for boron-doped porous carbons with varied microporosity and boron concentration. It was found out that pyrolysis temperature significantly determines the textural features, elemental composition, and CO_2_ adsorption capacity. With a narrow micropore volume of 0.27 cm^3^/g and a boron concentration of 0.79 at.% the representative adsorbent presents the maximum CO_2_ adsorption (2.51 mmol/g at 25 °C, 1 bar) and a CO_2_/N_2_ selectivity of 18 in a 10:90 (*v*/*v*) ratio. Last but not least, the as-prepared B-doped carbon adsorbent possesses a remarkable cyclic stability over five cycles, fast kinetics (95% equilibrium in 6.5 min), a modest isosteric heat of adsorption (22–39 kJ/mol), and a dynamic capacity of 0.80 mmol/g under simulated flue gas conditions. This study serves as a valuable reference for the fabrication of B-doped carbons using an environmentally benign activating agent for CO_2_ adsorption application.

## 1. Introduction

Driven mostly by industrial activities and the continuous dependence on fossil fuels, the constant increase in atmospheric carbon dioxide (CO_2_) levels (up to 421 ppm) has sharpened the worldwide need for creative and effective carbon capture and storage (CCS) technologies [1,2]. As a first step, CO_2_ capture from flue gas, or pre-combustion capture, is a primary step to stop the release of CO_2_ into the atmosphere. Among the many materials such as porous polymers [3,4,5], porous carbons [6,7,8,9,10], zeolites [11] and metal–organic frameworks (MOFs) [12,13] investigated for this use, porous carbon-based sorbent has shown promise because of the strong physicochemical stability, customizable pore shape, large surface area, and advanced adsorption capacity [14,15,16]. Conventional activating agents such as KOH, H_3_PO_4_, and ZnCl_2_ are widely used in the synthesis of porous carbons due to their strong activation capabilities [17,18]. However, their high corrosiveness, hazardous nature, and potential environmental impact present significant challenges in large-scale applications. The use of these aggressive pore-generated agents can lead to equipment degradation, the generation of toxic waste, and safety risks during handling and disposal. Therefore, the development of alternative, eco-friendly activating agents is crucial for promoting sustainable carbon material synthesis. In this context there are significant efforts shown to produce porous carbon using sustainable chemical activation strategies. For instance, Wang et al. reported that weak, corrosive K_2_CO_3_ activation enhances porosity, and the resulting adsorbent achieved a CO_2_ capture capacity of 4.36 mmol/g with good stability [19]. Also, D-glucose was carbonized in a one-step process at 800 °C using potassium acetate, producing porous carbon spheres with an SSA of 1917 m^2^/g and a specific pore volume of 0.85 cm^3^/g, achieving a CO_2_ capture capacity of 6.62 mmol/g at 0 °C and 1 bar [20]. Moreover, shrimp shells were processed too using sodium thiosulfate as an activating agent, yielding ternary (N, S, O)-doped porous carbon with an a CO_2_ adsorption capacity of 236.80 mg/g and a CO_2_/N_2_ selectivity of 84.3 [21]. In addition, in our previous studies we applied sodium thiosulfate (Na_2_S_2_O_3_) [22], potassium thiosulfate K_2_S_2_O_3_ [23], and potassium persulfate [24] as green and less corrosive chemical activating agents. However, to the best of our knowledge there are not any studies using sodium metaborate tetrahydrate (NaBO_2_·4H_2_O) as a green activating agent. Based on the MSDSs of KOH and NaBO_2_·4H_2_O in Figure S1 (Supplementary Materials), KOH is highly corrosive, causing severe burns, eye damage, and respiratory irritation. KOH is highly reactive with acids and moisture, while NaBO_2_·4H_2_O is more stable, with a lower instability rating. This approach not only minimizes hazardous by-products but also enhances the sustainability of porous carbon production by using NaBO_2_·4H_2_O as a pore forming agent.

Recent studies have shown that modifying carbon surfaces with heteroatoms significantly enhances CO_2_ affinity and selectivity through Lewis’s acid–base interactions [25,26,27,28]. Among the various heteroatoms used, nitrogen and sulfur are the most common due to their ability to introduce active sites and alter the electronic properties of the carbon matrix. This modification strengthens the interaction between CO_2_ molecules and the carbon surface, improving overall adsorption efficiency [29,30,31,32,33]. However, boron doping remains less explored despite its potential to enhance CO_2_ capture by interacting with the Lewis acid regions of CO_2_ molecules [34]. In addition to heteroatom doping, the presence of small micropores (<1 nm) further enhances CO_2_ adsorption by increasing van der Waals forces. This synergistic effect has sparked growing interest in developing biomass-derived porous carbons that integrate optimal porosity with strategic heteroatom incorporation. Such materials offer a sustainable and eco-friendly solution for addressing rising CO_2_ emissions.

In this regard, agricultural waste offers a plentiful and often underappreciated supply of high-performance carbon sorbents [35,36,37,38]. One particularly interesting option is the lignocellulosic residue of the water chestnut shell (WCS). Commonly discarded and generated in large quantities in regions where water chestnuts are cultivated—especially in Asia and parts of Europe—WCSs contributes to local waste accumulation and environmental burden. Comprising cellulose, hemicellulose, and lignin, its rich composition offers a naturally porous structure that, with heat processing, may be converted into a carbon-rich scaffold perfect for CO_2_ collection [39]. Selecting water chestnut shell as a forerunner fits the ideas of the circular economy as it transforms an unused waste into a useful instrument for reducing climate change. Moreover, its natural structural characteristics and chemical composition provide a special chance to design materials with customized porosity and usefulness, thereby differentiating it from traditional carbon sources such as coal or synthetic polymers [40].

To address these challenges, the aim of this work is to develop a sustainable and highly efficient boron-doped porous carbon adsorbent derived from water chestnut shell (WCS), using sodium metaborate tetrahydrate (NaBO_2_·4H_2_O) as a novel, green activating agent. This study seeks to elucidate the role of boron doping and narrow microporosity on CO_2_ capture performance, and to demonstrate the potential of WCS as a viable biomass precursor for scalable carbon material production.

Herein, the WCS has been utilized to synthesis boron-doped porous carbons using a two-stage process: first the pre-carbonization of WSCs and then the activation of biochar with sodium metaborate tetrahydrate (NaBO_2_·4H_2_O) at elevated temperatures. The resulting materials demonstrate a remarkable potential for CO_2_ adsorption. These results highlight how closely narrow microporosity, boron doping, and activation conditions interact to control performance. Beyond its capture efficiency, the material shows robustness over many cycles, fast adsorption kinetics, and modest energy needs for regeneration qualities that improve its practical practicality. By using WCSs, this work not only advances the development of environmentally friendly CO_2_ sorbents but also clarifies the possibility of agricultural wastes to significantly support carbon management strategies, opening the path for more research on biomass-based solutions in the struggle against climate change.

## 2. Results and Discussion

### 2.1. Morphology, Structural Phases, and Surface Chemistry Analysis

**Figure 1:**
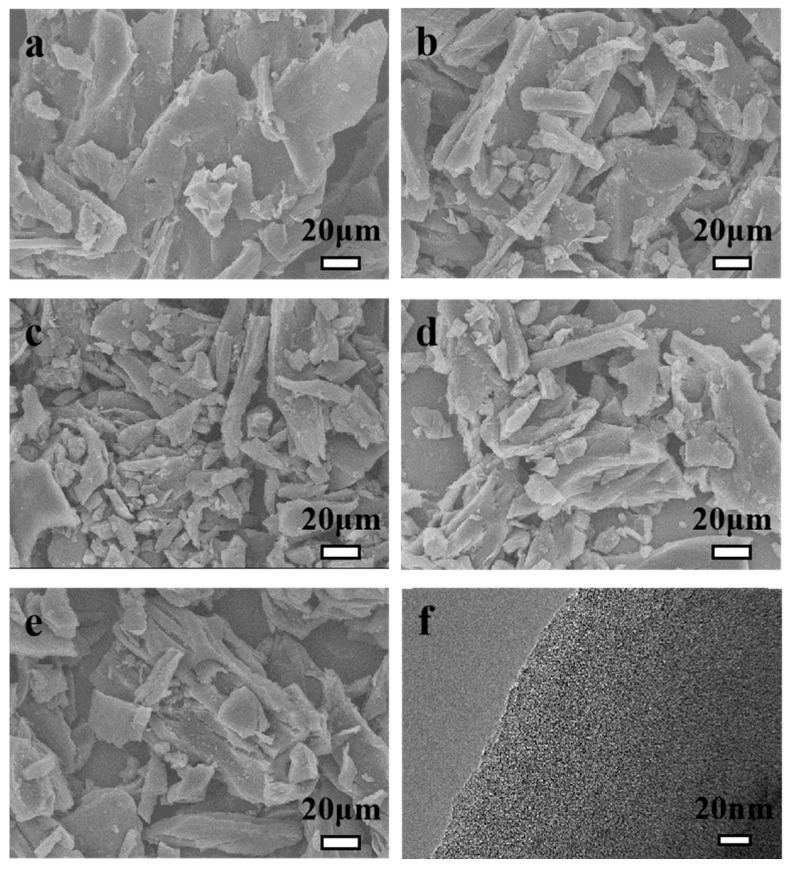
SEM images of (**a**) WSC, (**b**) WSCSM-650, (**c**) WSCSM-700, (**d**) WSCSM-750, and (**e**) WSCSM-800 and (**f**) TEM image of WSCPM-750.

The SEM images illustrate the morphological transformation of carbonized water chestnut shell (WSC) before and after activation with NaBO_2_·4H_2_O. Figure 1a presents the SEM image of WSC, which exhibits a relatively compact, layered structure with irregularly shaped carbon sheets. After activation, as shown in Figure 1b–e, the morphology undergoes significant changes, leading to a more fragmented structure with increased porosity. The activation process induces the formation of interconnected pores and a rougher surface texture, which enhances the surface area and facilitates gas adsorption. Figure 1f is a TEM image of a representative sample WSCSM-750, providing further insight into the nanoscale structure of the activated carbon. A distinctive wormhole-like microstructure is observed, indicating the formation of an intricate network of pores that can improve gas diffusion and adsorption efficiency. Additionally, the absence of well-defined crystalline domains in the TEM image confirms the amorphous nature of the carbon, suggesting that the activation process has effectively disrupted any ordered graphitic structures. This disordered arrangement contributes to enhanced adsorption properties by introducing more active sites and increasing the material’s overall surface area.

**Figure 2:**
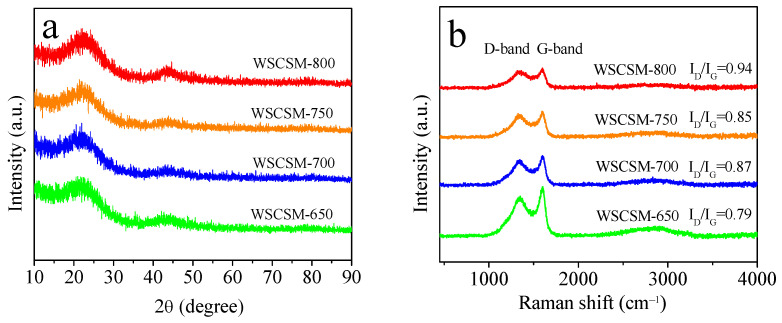
(**a**) XRD patterns and (**b**) Raman spectrum graph of B-doped porous carbons derived from WSC.

The structural characteristics of WSCSM-T samples were further analyzed using X-ray diffraction (XRD, Philips, Almelo, Holland) carried out on a PHILIPS PW3040/60 powder diffractometer using CuKα radiation (λ = 0.15406 nm) and Raman spectroscopy (Reneishaw, Gloucestershire, United Kingdom), examined with a Reneishaw InVia Raman spectrometer using laser excitation wavelengh at 532 nm. The XRD patterns (Figure 2a) exhibit a broad peak at approximately 23.2° corresponding to the (002) plane of carbon, and another wide peak around 43.6° which is assigned to the (100) plane [41,42]. The diffuse nature of these peaks confirms the amorphous structure of the prepared carbons, indicating a lack of long-range order. This observation aligns with the TEM image of WSCSM-750 (Figure 1f), where the absence of well-defined crystalline domains further supports the presence of an amorphous carbon framework. Raman spectroscopy (Figure 2b) provides further insight into the structural evolution of the activated carbons. The spectra display two characteristic peaks of the D-band at approximately 1358 cm^−1^, associated with structural defects and disorder, and of the G-band at around 1585 cm^−1^, corresponding to sp^2^-bonded graphitic carbon. The intensity ratio I_D_/I_G_ increases from 0.79 for WSCSM-650 to 0.94 for WSCSM-800, indicating a rise in disorder within the carbon framework as the activation temperature increases [43,44]. This trend suggests that the activation process progressively disrupts graphitic domains, creating more defect sites and enhancing porosity. The XRD, Raman, and TEM findings collectively demonstrate that NaBO_2_·4H_2_O activation effectively modifies the carbon structure by introducing porosity and disorder. The wormhole-like microstructure observed in TEM indicates the presence of hierarchical porosity, while the increasing I_D_/I_G_ ratio reflects a more defect-rich carbon matrix at higher activation temperatures. These structural modifications are expected to improve gas diffusion and adsorption capacity, making the WSCSM-T materials promising candidates for CO_2_ capture applications.

**Figure 3:**
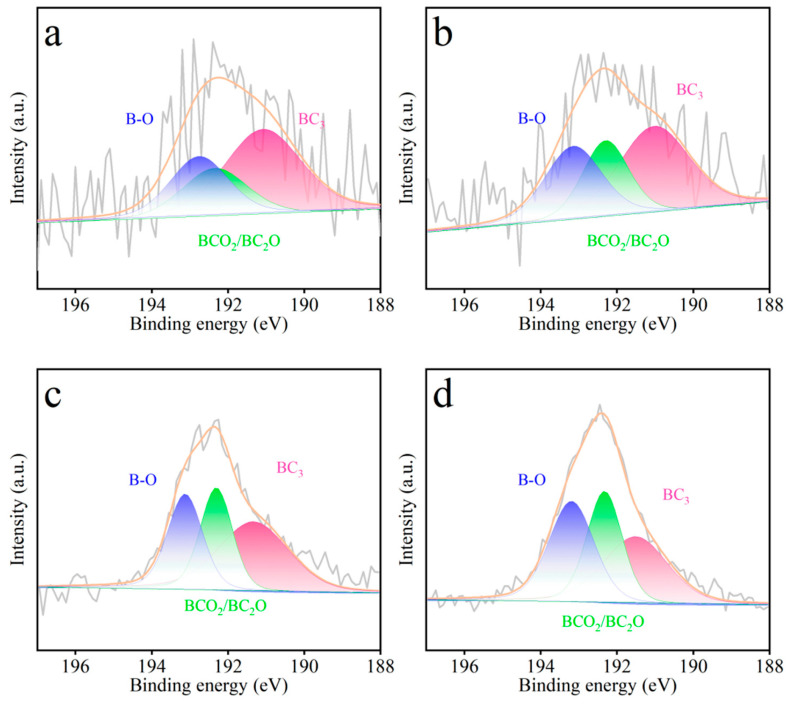
XPS analysis of B 1s spectrum images of (**a**) WSCSM-650, (**b**) WSCSM-700, (**c**) WSCSM-750, and (**d**) WSCPM-800.

X-ray photoelectron spectroscopy (XPS) was employed to investigate the surface chemistry and elemental composition of the WSCSM-T adsorbents, providing insights into the effectiveness of boron doping. The B 1s XPS spectrum of B-doped porous carbons (Figure 3) exhibits the following three distinct peaks corresponding to different boron bonding states: BC_3_ at 191.2 eV, BCO_2_/BC_2_O at 192.3 eV, and B–O at 193.1 eV. These peaks confirm the successful incorporation of boron into the carbon framework, primarily in the form of boron–carbon and boron–oxygen functionalities [34,45]. Quantitative XPS analysis (Table 1) reveals a progressive increase in boron content from 0.29 at% in WSCSM-650 to 1.65 at% in WSCSM-800. This trend suggests that higher activation temperatures facilitate enhanced boron incorporation, likely due to increased reactivity between the carbon matrix and the NaBO_2_·4H_2_O precursor. Notably, the presence of oxygen-bound boron species (BCO_2_/BC_2_O and B–O) indicates that boron is not only embedded within the carbon structure but also interacts with surface oxygen groups, potentially influencing the material’s electronic properties and CO_2_ adsorption performance. The dual role of NaBO_2_·4H_2_O, acting as both a pore-forming agent and a boron source, is evident from these results. Its contribution to porosity enhancement, combined with the introduction of boron functionalities, likely improves the interaction between the carbon surface and CO_2_ molecules. The increasing boron content at elevated temperatures may also contribute to modifications in the electronic structure of the carbon framework, further optimizing its adsorption capabilities. These findings underscore the significance of controlled boron doping in tuning the physicochemical properties of porous carbons for gas capture applications.

### 2.2. Textural Properties Analysis

**Figure 4:**
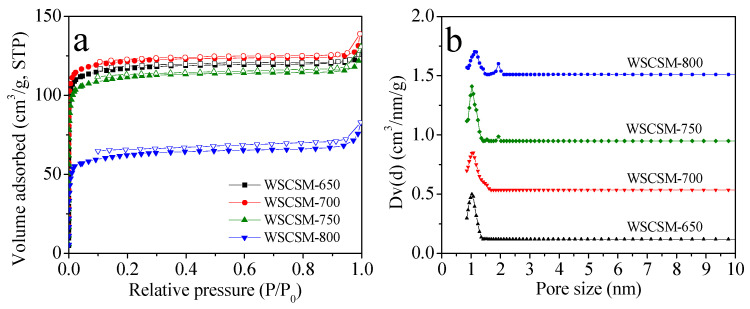
(**a**) Nitrogen adsorption–desorption isotherms and (**b**) pore size distribution of samples prepared under various conditions. In (**a**), filled symbols denote the adsorption branches, whereas empty symbols indicate the desorption branches.

The porous textural properties of WSCSM-T were evaluated using N_2_ adsorption/desorption measurements at −196 °C. As shown in Figure 4a, the sorption isotherms exhibit a distinct Type I behavior, characterized by a steep rise in adsorption at low relative pressures (P/P_0_ < 0.01), confirming the predominantly microporous nature of the WSCSM-T samples. The semi-plot of the N_2_ isotherm for WSCSM-T samples is shown in Figure S2 (Supplementary Materials). The textural parameters, including the specific surface area (S_BET_), total pore volume (V_0_), and micropore volume (V_t_) are summarized in Table 1. With increasing activation temperature, the porosity development follows a distinct trend. WSCSM-700 exhibits the highest S_BET_ (481 m^2^/g) and the largest micropore volume (V_0_ = 0.21 cm^3^/g), indicating that 700 °C is the optimal activation temperature for maximizing microporosity. However, further increasing the activation temperature to 750 °C and 800 °C results in a decline in both surface area and pore volume. Specifically, WSCSM-800 undergoes a significant reduction in S_BET_ to 228 m^2^/g and a corresponding decrease in micropore volume (V_0_ = 0.12 cm^3^/g). This decrease is attributed to structural degradation and pore collapse at elevated temperatures, a phenomenon commonly observed in highly porous carbon materials subjected to excessive thermal treatment. Narrow micropores (<1 nm) play a crucial role in determining CO_2_ adsorption performance under low-pressure conditions. To assess the contribution of these ultra-micropores, the narrow micropore volumes (V_n_) were estimated using the Dubinin–Radushkevich (D–R) equation based on CO_2_ adsorption data. As listed in Table 1, V_n_ values range from 0.24 to 0.27 cm^3^/g, following a trend similar to that observed for S_BET_ and V_t_. The pore size distribution (Figure 4b), derived from the density functional theory (DFT) model, provides further insight into the structural characteristics of the WSCSM-T samples. All samples exhibit a dominant micropore size distribution in the range of 1–2 nm, reinforcing their classification as microporous materials. However, with increasing activation temperature, the development of mesopores becomes more apparent, particularly in WSCSM-750 and WSCSM-800. This transition towards a hierarchical pore structure suggests that excessive activation can lead to partial pore widening and the formation of secondary mesopores, which may influence gas diffusion and adsorption kinetics. Overall, these findings highlight the intricate balance between activation temperature and porosity development, where moderate activation temperatures (around 700 °C) favor micropore formation, whereas higher temperatures (≥750 °C) can lead to structural degradation and mesopore generation. The resulting pore architecture is crucial for optimizing CO_2_ adsorption performance and diffusion efficiency in porous carbon materials.

### 2.3. CO_2_ Adsorption Performance

**Figure 5:**
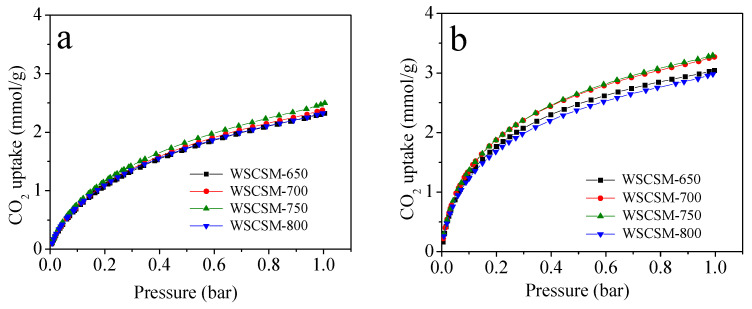
CO_2_ adsorption isotherms for B-doped porous carbons derived from WSC. The isotherms are shown at two distinct temperatures: (**a**) 25 °C and (**b**) 0 °C.

The CO_2_ adsorption performance of WSCSM-T samples was evaluated at 25 °C and 0 °C, as shown in Figure 5. The adsorption capacities of the samples vary with changes in textural properties and boron content, highlighting the intricate relationship between porosity and surface chemistry in determining CO_2_ uptake. Narrow micropores (<1 nm) play a dominant role in CO_2_ adsorption under low pressure due to their strong interactions with CO_2_ molecules via physisorption. As V_n_ increases, the CO_2_ adsorption capacity rises from 2.32 to 2.51 mmol/g at 25 °C and 1 bar which confirms the crucial role of these micropores in enhancing adsorption performance. At 0.15 bar, the typical partial CO_2_ pressure in post-combustion flue gas, the maximum CO_2_ uptake for these B-doped porous carbons was 0.98 and 1.65 mmol/g at 25 and 0 °C and 1 bar, respectively. Notably, WSCSM-700 and WSCSM-750 have the same V_n_ value, yet WSCSM-750 exhibits higher CO_2_ uptake, likely due to its greater boron content. Boron doping enhances CO_2_ adsorption by introducing electron-deficient sites, thereby improving the carbon surface’s affinity for CO_2_ molecules. However, despite WSCSM-800 containing the highest boron content its adsorption capacity does not surpass that of WSCSM-700 or WSCSM-750. This suggests that while boron doping positively influences CO_2_ uptake it cannot fully compensate for the loss of narrow micropores, which remain the primary adsorption sites. These findings indicate that CO_2_ uptake is governed primarily by narrow microporosity, which provides high adsorption potential, and secondarily by boron doping, which enhances surface interactions. Therefore, optimizing both structural (narrow microporosity) and chemical (boron doping) properties is essential for maximizing CO_2_ capture efficiency.

It is worth mentioning that although the maximum CO_2_ uptake of WSCSM-T samples (2.51 mmol/g) is lower than that of some porous carbons derived from KOH activation [46,47,48,49] it remains comparable to many classical solid adsorbents, such as porous carbons [22,24,50], COFs [51], porous polymers [3], and MOFs [12], among others.

**Figure 6:**
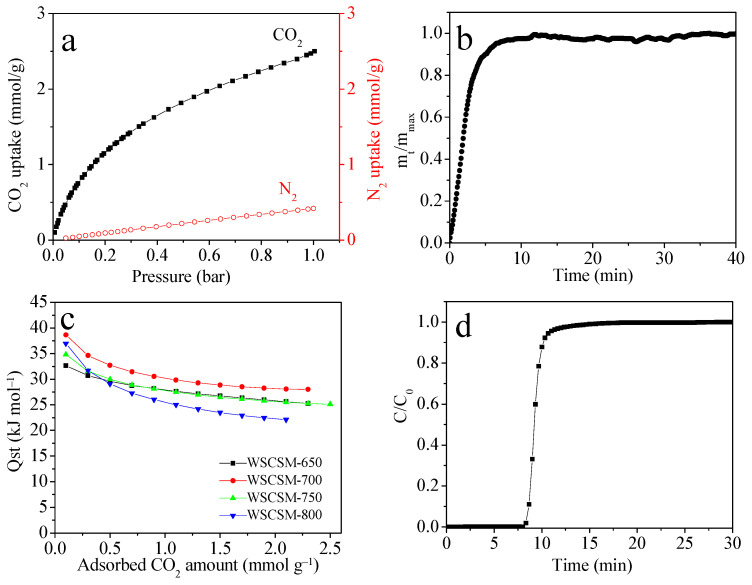
(**a**) CO_2_ and N_2_ adsorption isotherms for WSCSM-750 measured at 25 °C and 1 bar, (**b**) CO_2_ adsorption kinetics at 25 °C for WSCSM-750, (**c**) isosteric heat of CO_2_ adsorption (Q_st_) on WSCSM-T adsorbents derived from experimental adsorption isotherms at 0 °C and 25 °C, and (**d**) breakthrough curves of WSCSM-750 under the following conditions: adsorption temperature of 25 °C, gas flow rate of 10 mL/min, inlet CO_2_ concentration of 10 vol.%, and gas pressure of 1 bar.

Furthermore, Figure 6 provides a comprehensive evaluation of the CO_2_ adsorption behavior and separation performance of WSCSM-T samples. Figure 6a presents the CO_2_ and N_2_ adsorption isotherms of WSCSM-750 at 25 °C and 1 bar. The material exhibits significantly higher CO_2_ uptake compared to N_2_, indicating a strong affinity toward CO_2_. This enhanced selectivity can be attributed to the combination of an ultra-microporous structure and heteroatom-doped surface chemistry. CO_2_ has a larger quadrupole moment (–13.4 × 10⁻^40^ C·m^2^) and higher polarizability than N_2_ (–4.7 × 10⁻^40^ C·m^2^), which makes it more responsive to electrostatic interactions and Lewis basic sites introduced by boron doping. In addition, the smaller kinetic diameter of CO_2_ (3.3 Å vs. 3.64 Å for N_2_) allows it to access narrow micropores more effectively, further contributing to the observed selectivity. As a result, the ideal adsorption solution theory (IAST) [52] predicts a CO_2_/N_2_ (10:90, *V*/*V*) selectivity of 18, demonstrating the potential of WSCSM-750 for efficient gas separation applications.

The adsorption kinetics curve (Figure 6b) reveals rapid CO_2_ adsorption, with 95% of the equilibrium capacity achieved within 6.5 min. This fast uptake suggests that WSCSM-750 possesses a well-developed porous structure and high surface accessibility, facilitating efficient gas diffusion. Such rapid adsorption kinetics make WSCSM-750 a strong candidate for cyclic adsorption–desorption processes, which are crucial for reducing energy consumption in industrial CO_2_ capture and regeneration applications.

Figure 6c illustrates the isosteric heat of adsorption (Q_st_) for CO_2_ on WSCSM-T adsorbents, calculated using the Clausius–Clapeyron equation based on isotherm data at 0 °C and 25 °C. The Q_st_ values range from 33 to 39 kJ/mol at near-zero loading, with an overall range of 22–39 kJ/mol. These values suggest that CO_2_ adsorption is predominantly governed by physisorption, ensuring good regenerability without significant loss of capacity. The relatively high Q_st_ at low coverage also indicates the presence of energetically favorable adsorption sites, likely influenced by heteroatom doping or specific structural features of WSCSM-750.

The breakthrough curve (Figure 6d) further supports the material’s practical applicability, demonstrating a dynamic CO_2_ capture capacity of 0.80 mmol/g under realistic flow conditions (25 °C, 10 mL/min flow rate, 10 vol.% CO_2_, and 1 bar total pressure). The combination of fast kinetics, high selectivity, moderate adsorption heat, and stable dynamic capacity underscores WSCSM-750 as a promising and practical adsorbent for CO_2_ separation processes. It needs to be stated here that there is a certain amount of water vapor present in practical flue gas, which could deteriorate the CO_2_ capture capacity of these B-doped carbons due to the competitive adsorption between CO_2_ and H_2_O.

**Figure 7:**
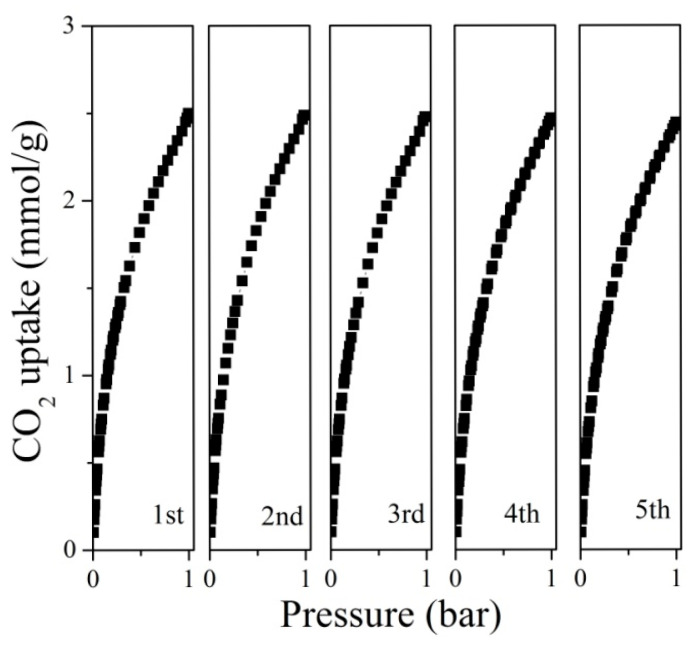
Cyclic study of CO_2_ adsorption for WSCSM-750.

The cyclic stability of WSCSM-750 as a CO_2_ sorbent was evaluated over five successive adsorption–desorption cycles (Figure 7). Before each test the sample was heated at 200 °C for 6 h in a vacuum. The results demonstrate remarkable stability, with no significant decrease in adsorption capacity throughout the cycles. This outstanding uniformity highlights the material’s excellent reusability and durability, making it a promising candidate for long-term CO_2_ capture applications. The sustained performance over multiple cycles suggests that WSCSM-750 maintains its structural integrity and adsorption sites, preventing pore blockage or degradation. This stability is essential for industrial applications, where repeated adsorption and regeneration cycles are required for cost-effective and energy-efficient CO_2_ capture.

## 3. Synthesis and Characterization

The dried water chestnut shell was pulverized into a fine powder and subsequently carbonized in a tube furnace. With a continuous N_2_ flow of 100 mL/min, approximately 5 g of the precursor was placed in a ceramic boat and heated to 500 °C at a rate of 5 °C/min. The preservation of a temperature of 500 °C for two hours ensured complete carbonization. The resultant carbonized product, referred to as WSC, was collected and stored for future use after cooling to ambient temperature in a nitrogen atmosphere.

Boron-doped porous carbons were synthesized through an activation process using NaBO_2_·4H_2_O as the activating agent. The WSC and NaBO_2_·4H_2_O were mixed in a 1:1 mass ratio, and activation was carried out at four different temperatures: 650, 700, 750, and 800 °C. The activated porous carbons were labeled as WSCSM-T, where T indicates the activation temperature. Detailed procedures for material preparation, physical characterization, and CO_2_ analysis are provided in the Supplementary Materials.

## 4. Conclusions

This study successfully synthesized boron-doped porous carbons from water chestnut shells through carbonization and NaBO_2_∙_2_O activation at temperatures ranging from 650 to 800 °C. Among the resultant WSCSM-T materials, WSCSM-750 emerged as the optimal sorbent, exhibiting a high narrow micropore volume (0.27 cm^3^/g) and moderate boron doping (0.79 at%). These structural and chemical features enabled WSCSM-750 to achieve a CO_2_ adsorption capacity of 2.51 mmol/g at 25 °C and 1 bar, a CO_2_/N_2_ selectivity of 18, fast adsorption kinetics, and a dynamic CO_2_ capture capacity of 0.80 mmol/g. The activation temperature was identified as a crucial factor in balancing narrow microporosity and boron incorporation, which together govern CO_2_ adsorption performance. These findings underscore the dual role of NaBO_2_∙4H_2_O as both an activating and doping agent and highlight the potential of water chestnut shells as a sustainable precursor for high-performance CO_2_ sorbents. Furthermore, WSCSM-750 demonstrated excellent cyclic stability and moderate *Q_st_* values, indicating a well-balanced adsorption strength that ensures both efficient CO_2_ capture and easy regenerability.

Future research could explore co-doping with additional heteroatoms or fine-tuning activation conditions to further enhance CO_2_ selectivity and capacity. This study paves the way for scalable, biomass-derived solutions to mitigate CO_2_ emissions, contributing to the development of sustainable carbon capture technologies.

## Figures and Tables

**Table 1:** Porous properties, elemental compositions, and CO_2_ uptakes of precursors and B-doped sorbents derived from different conditions.

Sample	S_BET_^a^ (m^2^/g)	V_0_^b^ (cm^3^/g)	V_t_^c^ (cm^3^/g)	V_n_^d^ (cm^3^/g)	XPS (at. %)				CO_2_ Uptake (mmol/g)	
					C	N	B	O	25 °C	0 °C
WSC	116	0.10	0.01	0.19	1.18	84.67	0.57	13.57	1.56	2.11
WSCSM-650	464	0.19	0.18	0.24	2.30	87.62	0.29	9.78	2.32	3.05
WSCSM-700	481	0.21	0.19	0.27	0.97	90.46	0.40	8.17	2.38	3.27
WSCSM-750	437	0.19	0.15	0.27	0.95	88.43	0.79	9.83	2.51	3.30
WSCSM-800	228	0.12	0.07	0.26	0.78	86.33	1.65	11.25	2.33	2.98

^a^ Surface area was calculated using the BET method at P/P_0_ = 0.001–0.01. ^b^ Total pore volume at P/P_0_ = 0.99. ^c^ Evaluated by the t-plot method. ^d^ Pore volume of narrow micropores (<1 nm) obtained from the CO_2_ adsorption data at 0 °C.

## Data Availability

The data presented in this study are available on request from the corresponding author.

## Supplementary Materials

The following supporting information can be downloaded at: https://www.mdpi.com/article/10.3390/molecules30122564/s1, Scheme S1. Schematic diagram of the fixed-bed reactor system, Figure S1: Material safety data sheets (MSDSs) of KOH and NaBO_2_·4H_2_O. Figure S2: Semi-plot of the N_2_ isotherm for WSCSM-T samples.
