# Supplementary material for: The Synthesis of B-Doped Porous Carbons via a Sodium Metaborate Tetrahydrate Activating Agent: A Novel Approach for CO2 Adsorption
Source: Molecules. 2025 Jun 12;30(12):2564. doi: 10.3390/molecules30122564 (PMC12195970; doi:10.3390/molecules30122564)
Supplement: Supplementary file 1 [file molecules-30-02564-s001.zip › molecules-3671706-supplementary.pdf]

Synthesis of B-Doped Porous Carbons via Sodium Metaborate Tetrahydrate Activating Agent: A Novel Approach for CO_2 Adsorption

(Supplementary materials)

Junting Wang[1], Yingyi Wang[1], Xiaohan Liu[1], Qiang Xiao[2], Muslum Demir[3,4], Mohammed K. Almesfer[5], Suleyman Gokhan Colak[6], Linlin Wang[7], Xin Hu[1,*], Ya Liu[1,*]

[1] Key Laboratory of the Ministry of Education for Advanced Catalysis Materials, Zhejiang Normal University, Jinhua, Zhejiang, 321004, PR China

[2] Institute of Plant Nutrition, Resources and Environment, Beijing Academy of Agriculture and Forestry Science. Beijing 100097, PR China

[3] Department of Chemical Engineering, Bogazici University, 34342 Istanbul, Türkiye

[4] TUBITAK Marmara Research Center, Material Institute, Gebze 41470 , Türkiye

[5] Chemical Engineering Department, College of Engineering, K. S. A & Central labs, King Khalid University, Abha 61413, Saudi Arabia.

[6] Department of Biomedical Engineering, Faculty of Engineering and Natural Sciences, Iskenderun Technical University, Hatay 31200, Türkiye

[7] Key Laboratory of Urban Rail Transit Intelligent Operation and Maintenance Technology and Equipment of Zhejiang Province, College of Engineering, Zhejiang Normal University, Jinhua, Zhejiang, 321004, PR China

[*] Corresponding author's e-mail: huxin@zjnu.cn (X. H.) and sky48@zjnu.cn (Y. L.); phone: 86-151-0579-0257; fax: 86-579-8228-8269

Carbonization

Water chestnut shell to be used as a precursor have been cleaned, crushed, and sieved into the particles in the size range of 100–140 mesh (105–150 μm). Carbonization was performed in a horizontal quartz tubular reactor under a nitrogen flow (using a flow rate of 200 mL/min). The samples were heated from room temperature to 500 °C with a heat rate of 5 °C/min. The samples were maintained at

the final temperature for 2 h and then cooled in the nitrogen atmosphere. To simplify, carbonized water chestnut shell was denoted as WSC.

$NaBO_2 \cdot 4H_2O$ activation

For a typical reaction, 2 g WSC was combined with a solution that contained 2g $NaBO_2 \cdot 4H_2O$. After stirring vigorously for 6 h, the mixture was left overnight to dry at 120 °C in an oven. Afterwards, the sample was activated to 750 °C for 2 h. During the activation process, the heating rate is 5 °C/min and nitrogen flow rate is 400 mL/min. Following activation, the sorbent was rinsed with distilled water until the pH value of the filtrate was roughly 7. The wet sample was then dried at 150 °C under vacuum for 24 h. The obtained sample was denoted as WSCSM-750-2.

Characterization

Powdered X-ray diffraction (XRD) patterns were carried out on a PHILIPS PW3040/60 powder diffractometer using CuKα radiation (λ =0.15406nm). Scanning electron microscopy (SEM Hitachi S-4800) was used to observe the morphology of the samples of carbon materials. Further details of the pore structure were determined by transmission electron microscopy (TEM, JEOL-2100F) operated at 200 kV. Nitrogen adsorption and desorption isotherms were measured on a Beishide 3H-2000PS2 sorption analyzer at -196ºC. Ultrahigh-purity N_2 (99.999%, Shanghai Pujiang Gas Co., Ltd) was used for measurement. Before measurement, the samples were degassed in a vacuum at 200ºC for at least 12h. The specific surface area (S_{BET}) was calculated according to the multipoint Brunauer-Emmett-Teller (BET) method from the adsorption data in the relative pressure range between 0.001 and 0.01. The total micropore volume (V_t) was deduced from the N_2 adsorption data by the t-plot method, and the total pore volume (V_0) was estimated from the adsorbed amount of liquid nitrogen at a relative pressure of 0.99. The error of porosity measurement is

within 3%. The pore size distribution was calculated using the density functional theory (DFT) method. In addition, X-ray photoelectron (XPS) measurements were performed using an AXIS Nova spectrometer (Kratos Inc., NY, USA) equipped with a monochromatic Al Kα X-ray source (1486.6 eV). XPS survey spectra were recorded with a pass energy of 160 eV, and high-resolution spectra with a pass energy of 40 eV.

The CO_2 adsorption isotherms were measured using the Beshide 3H-2000PS2 sorption analyzer at 0ºC and 25ºC, respectively. Pure CO_2 (99.99%, Shanghai Pujiang Gas Co., Ltd) was used for adsorption. Prior to each adsorption experiment, the sample was degassed for 12 h at 200ºC to remove the guest molecules from the pores. The volume of narrow micropores (with sizes <1 nm), V_n, was calculated from CO_2 adsorption at 0°C using the Dubinin–Radushkevich (D-R) equation. The measurements were repeated for each sample, until the values fell within ± 2% of each other.

Measurement of dynamic CO_2 uptake of the sorbents

The dynamic CO_2 uptake of the sorbents was tested on a fixed-bed reactor schematically illustrated in Scheme S1 at 1 bar and 25 °C. First, sample was heated at 100°C for 1 h under N_2 at a flow rate of 20 mL/min. The gas flow was shifted from nitrogen to a 10% mixture of CO_2 in N_2 at a flow rate of 10 mL/min, when the sample temperature was lowered to 25°C. The effluent gases were monitored online using an Agilent 7820A gas chromatograph with a thermal conductivity detector (TCD) to obtain the breakthrough curve.

The dynamic CO_2 uptake of the sorbent (q_d) was calculated from the breakthrough curves using the following equation:

$$q_d = \frac{Q_F C_0 t_s}{w} \qquad (1)$$

In the above equation, Q_F is the feed molar flow rate, C_0 is the concentration of the adsorbate in the feed stream, w is the weight of the adsorbent materials loaded in the column and t_s is the stoichiometric time, which can be estimated from breakthrough curves using the equation below:

$$t_s = \int_0^\infty \left(1 - \frac{C_A}{C_0}\right) \partial t \qquad (2)$$

Where C_A is the adsorbate concentration at the column outlet.

Measurement of CO_2 adsorption kinetics

The adsorption kinetics of CO_2 was measured in a thermogravimetric analyzer (NETZSCH STA 449C). In the kinetic analysis, the sample (~5 mg) was degassed under a He stream at 200°C for 1 h. Next, the temperature was cooled to the experimental temperature of 25°C. Then the CO_2 gas was fed into the test chamber with a flow rate of 50 mL/min and the weight variation with time was recorded. The measurement' s error is within 2%.

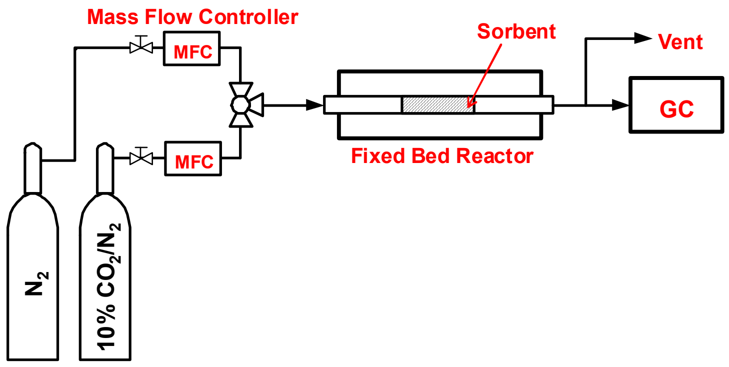

Scheme S1. Schematic of the fixed-bed reactor system.

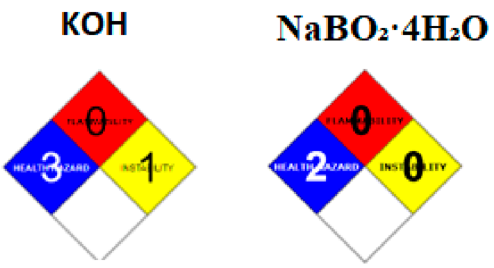

Figure S1: Material Safety Data Sheet (MSDS) of KOH and $NaBO_2 \cdot 4H_2O$, Sources:

https://studylib.net/ and https://datasheets.scbt.com/

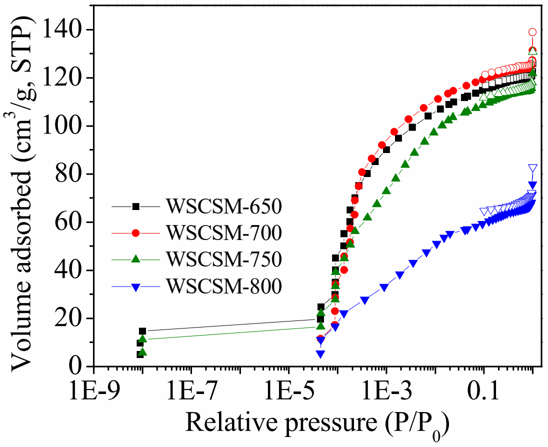

Figure S2. Semi-plot of the N_2 isotherm for WSCSM-T samples. Filled symbols denote the adsorption branches, whereas empty symbols indicate the desorption branches.
